# Endoscopic Retrograde Cholangio-Pancreatography and Endoscopic Ultrasound in the Management of Paediatric Acute Recurrent Pancreatitis and Chronic Pancreatitis

**DOI:** 10.3390/jcm13185523

**Published:** 2024-09-18

**Authors:** Deepak Joshi, Taimur Shafi, Usama Al-Farsi, Margaret G. Keane, Tassos Grammatikopoulos, Rania Kronfli, Erica Makin, Mark Davenport, Elizabeth Hayward, Andrew Pool, David Reffitt, John Devlin, Philip Harrison

**Affiliations:** 1 Institute of Liver Studies, King’s College Hospital NHS Foundation Trust, London SE5 9RS, UKdr-usama211@hotmail.com (U.A.-F.);; 2 Department of Gastroenterology, Nizwa Hospital, Oman; 3 Division of Gastroenterology and Hepatology, Johns Hopkins Hospital, Baltimore, MD 21287, USA; 4 Paediatric Liver, GI & Nutrition Centre and Mowat Labs, King’s College Hospital NHS Foundation Trust, London SE5 9RS, UK; 5 Department of Paediatric Surgery, King’s College Hospital NHS Foundation Trust, London SE5 9SR, UKerica.makin@nhs.net (E.M.);; 6 Department of Anaesthetics, King’s College Hospital NHS Foundation Trust, London SE5 9SR, UK

**Keywords:** chronic pancreatitis, paediatric, endoscopic retrograde cholangio-pancreatography, endoscopic ultrasound, pancreatic duct stenting

## Abstract

**Highlights:**

**What are the main findings?**

- CP and ARP are common in paediatric populations with varied aetiologies.
- ERCP is more commonly used compared to EUS in paediatrics and is limited to specialist centres.

**What is the implication of the main finding?**

- ERCP and EUS are safe and efficacious, with minimal complications in the management of CP and ARP in a paediatric cohort.
- Indications for both diagnostic and therapeutic EUS are increasing.
- ERCP and EUS offer a more ‘patient-friendly’, minimally invasive approach to CP and ARP.

**Abstract:**

**Objectives**: To evaluate the role of ERCP (endoscopic retrograde cholangio-pancreatography) and EUS (endoscopic ultrasound) and to describe the efficacy and safety of these procedures in a paediatric cohort with chronic pancreatitis (CP) and acute recurrent pancreatitis (ARP). **Methods**: All patients (<18 years) undergoing an ERCP or EUS for ARP and CP between January 2008 and December 2022 were included. Data collection included indications for the procedure, technical success, adverse events and outcome data. **Results**: A total of 222 ERCPs were performed in 98 patients with CP and ARP (60% female, median age 10 years). The commonest indications were a main pancreatic duct stricture (PD) with or without a stone within the main PD. Successful cannulation was achieved in 98% of cases. Improved stricture resolution was demonstrated in 63% of patients. The overall adverse event rate for ERCP was low (n = 8/222, 3.6%). An improvement in abdominal pain was demonstrated in (75/98) 76% of patients. Their Body Mass Index also significantly improved post ERCP (15.5 ± 1.41 vs. 12.9 ± 1.16 kg/m^2^, *p* = 0.001). A total of 54 EUS procedures were undertaken in 48 individuals. Moreover, 35 individuals underwent a therapeutic EUS procedure, for which the commonest indication was the drainage of a pancreatic fluid collection. The overall complication rate was low (n = 2.4%) in all EUS cases. **Conclusions**: ERCP and EUS can be safely and effectively used in a paediatric population with indications analogous to an adult cohort.

## 1. Introduction

Chronic pancreatitis (CP) and acute recurrent pancreatitis (ARP) in paediatric populations are typically caused by anatomical variants, i.e., pancreatic divisum, choledochal malformations, genetic mutations in cystic fibrosis transmembrane conductance regulator (CFTR), Protein Serine type 1 (PRSS1), serine protease inhibitor kazal type 1 (SPINK 1), chymotrypsin C (CTRC) and carboxy Discussion at an HPB MDT meetingtidase 1 (CPA1) genes, infections, autoimmune causes and trauma [1,2]. The estimated incidence of CP in Western populations varies between 0.5 and 2.0 per 100,000 children per year [2,3,4]. Although CP is deemed rare in children, it is often associated with significant and potentially debilitating symptoms, a failure to thrive, impaired growth, increased health care costs and a poor quality of life [1,5].

The INSPPIRE-2 (International Study Group of Paediatric Pancreatitis: In Searching for a Cure) study is the largest prospective cohort of paediatric patients with ARP or CP [1]. This study and its predecessor (INSPPIRE) have provided invaluable information for health care professionals, patients and their family members in determining risk factors and the burden of disease [6]. However, data on hepato-pancreatico-biliary (HPB) endoscopic interventions were limited.

A multi-disciplinary team approach is key in the management of individuals with CP, irrespective of age. HPB endoscopic techniques such as ERCP (endoscopic retrograde cholangio-pancreatography) and EUS (endoscopic ultrasound) are well-established diagnostic and therapeutic modalities in CP [7]. ERCP has been used for more than 30 years in paediatric patients with CP [8,9,10]. EUS provides a complimentary role, replacing many diagnostic ERCP examinations, as well as providing an alternative method for draining PFCs (pancreatic fluid collections), i.e., EUS-guided cyst gastrostomy and managing pancreatic strictures in failed ERCP examinations or in individuals with altered anatomy, i.e., EUS-guided pancreaticogastrostomy. The experience of ERCP and EUS in a UK paediatric population with CP and ARP is limited [8,11].

Therefore, the aim of this study was to evaluate the indications for and safety and efficacy of ERCP and EUS in a paediatric cohort with CP and ARP at a large UK tertiary referral centre.

## 2. Methods

### 2.1. Patients

This is a retrospective analysis of a prospectively collected dataset. All paediatric patients (<18 years of age) who underwent an ERCP or an EUS for CP or ARP at King’s College Hospital between the dates of 1 January 2008 and 31 December 2022 were included [12]. The diagnoses of ARP and CP were as per the INSPPIRE criteria [1]. Data collection included participant demographics, presenting symptoms, the aetiology of their pancreatitis and imaging. For each ERCP or EUS procedure indication, the intervention, rates of technical success, rates of adverse events (AEs) and outcomes were recorded, along with any subsequent surgical procedures, if required. This study was approved by an Institutional Review Board (IRAS ID 238002). All patient records were anonymized before analysis.

### 2.2. Endoscopic Procedures

Written informed consent was obtained for the procedures from the parent(s) or legal guardian(s) of all children prior to the procedures, as per the standard of care. All endoscopic procedures were performed under general anaesthetic (E.H. and A.P.) in the endoscopy department in the supine position by adult interventional endoscopists (D.J., D.R., J.D. and P.H.). Where indicated, patients received peri-procedural intravenous antibiotics. 

### 2.3. Endoscopic Retrograde Cholangio-Pancreatography

Procedures were performed using a paediatric or adult duodenoscope (JF or TJF; Olympus, UK), Most children older than 1 year (weighing > 10–15 Kg) tolerated a standard adult duodenoscope (TJF). All ERCP procedures were performed in the endoscopy unit with fluoroscopy. Sphincterotomy was performed using standard accessories (Dash sphincterotome, Cook Medical, or DreamTome, Boston Scientific, Hong Kong, China). GT-1T or GT-UT cannulas were used (Cook Medical). Stones were extracted with standard baskets or extraction balloons (Olympus or Cook Medical). The guidewires used included Terumo 0.035, 450 cm Europe, Hydra Jagwire^TM^ 0.035 cm, Boston Scientific, or 0.025 and 0.035 cm VisiGlide^TM^, Olympus. Plastic stents (7 Fr, 10 Fr Cotton-Leung, Cook Medical; 7 Fr Compass BDS, Cook Medical; 5 Fr, 7 Fr Zimmon, Cook Medical) or fully covered metal stents (8 mm or 10 mm Wallflex, Boston Scientific; 6 mm Niti-S Bumpy, Taewoong Medical) were placed at the discretion of the endoscopist. In 2019, the biodegradable Archimedes (AMG International, Q3 Medical) 6 Fr, 8 Fr, and 10 Fr stents (fast and slow degradation rates) became commercially available.

### 2.4. Endoscopic Ultrasound

For children >1 year of age, an Olympus linear or radial echoendoscope was used, whereas, for those <1 year of age, an endobronchial ultrasound (EBUS) was used. Fine needle aspiration (FNA) was performed using a 22–25 G Expect needle (Boston scientific) or a 22 G Sharkcore needle. Fine needle biopsies (FNBs) were performed using a 22 G or 19 G Sharkcore needle (Medtronic). For patients with a symptomatic pancreatic fluid collection (PFC), an EUS-guided cystgastrostomy was performed. In all cases, prior to draining, doppler was used to ensure there were no intervening vessels, and the collection was carefully evaluated to ensure it was less than 1 cm from the transducer. Draining was performed via a Lumen Apposing Metal Stent (LAMS, Hot AXIOS, Boston Scientific) or using double pigtail stents. In cases of double pigtail stents, the collection was first punctured with a 19-gauge needle. A track was then formed by advancing a 6 Fr cystotome (Cook Medical) over the wire. After that, Hydra Jagwire^TM^ 0.035-inch guidewires were then advanced into the PFC and allowed to coil within the cyst under fluoroscopic guidance. The tract was then dilated with a Hurricane RX (4–6 mm) wire-guided balloon (Boston Scientific) or Soehendra biliary dilator before the insertion of, typically, two double-pigtail stents (7F, 5–7 cm). The fluid collected was sent for microscopy, culture and sensitivity, Gram stain and amylase tests where indicated.

### 2.5. Follow-Up

Following the procedure, patients were recovered by a trained paediatric nurse in recovery before being discharged back to a specialist paediatric ward for at least 24 h of observation prior to discharge. Periprocedural adverse events (AEs) were recorded and graded according to the American Society for Gastrointestinal Endoscopy (ASGE) lexicon [13]. During the follow-up period, patients were typically seen every 3–6 months in a paediatric pancreatitis clinic. Any additional endoscopic procedures, surgeries or further interventions—including changes to analgesic medications—were recorded.

### 2.6. Statistical Analysis 

Statistical analysis was performed using Social Sciences for Windows, version 18.0 (SPSS Inc., Chicago, IL, USA). The associations between various clinical and radiographic characteristics were evaluated using a 2-sample *t* test for continuous variables and a chi-squared test for categorical variables. A *p* value < 0.05 is considered statistically significant. Variables are presented as mean ± standard deviation or median and range where appropriate.

## 3. Results

### 3.1. Demographics

Over the 16-year study period, 562 children (under the age of 18 years) underwent an ERCP (n = 486) or EUS (n = 76) procedure. A total of 111 (20%) patients had a diagnosis of CP and 25 patients had a diagnosis of ARP.

**Figure 1:**
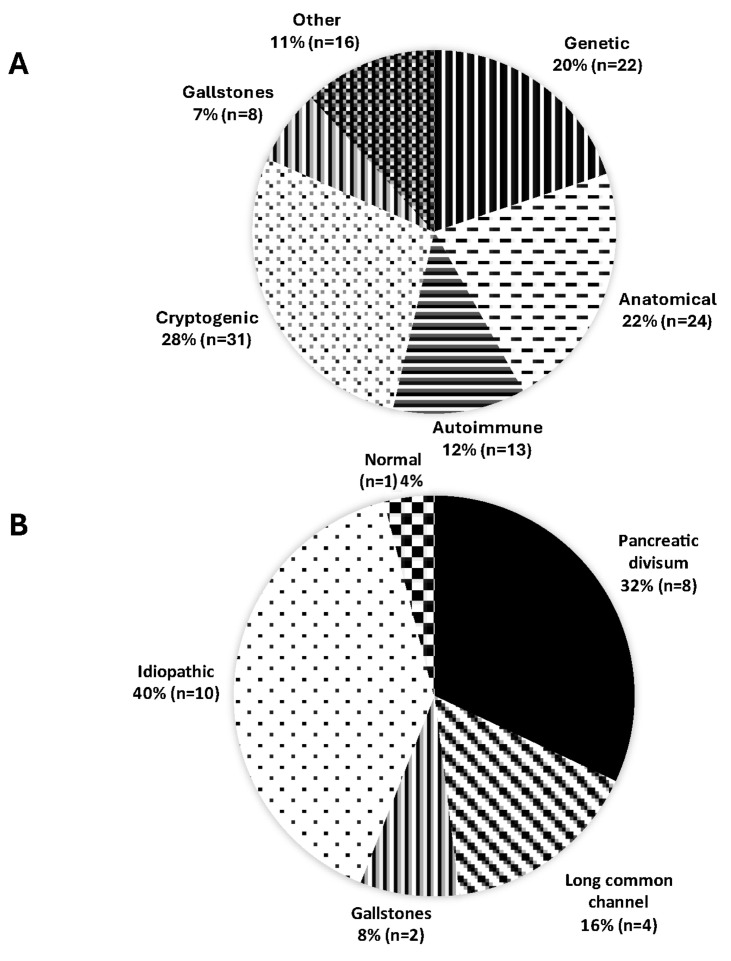
Aetiologies of (**A**) chronic pancreatitis and (**B**) acute recurrent pancreatitis.

In patients with CP or ARP, the commonest reported symptom was abdominal pain (93%, n = 127). Pancreatic exocrine insufficiency was present in 76% of individuals (n = 103). A total of 98 patients underwent an ERCP, 44 patients underwent an EUS, and 16 underwent combined ERCP and EUS. Most were female (n = 75, 55%), and the median age of the cohort was 11 (range 1–18) years. The common aetiologies of chronic pancreatitis included genetic (20%, n = 22), anatomical abnormality (22%, n = 24), autoimmune (12%, n = 13), gallstone (7%, n = 8), and cryptogenic (28%, n = 31) pancreatitis (Figure 1A). Of those with hereditary pancreatitis, variants in the PRSS1 and SPINK1 genes were the most prevalent (16/22, 74% of individuals). Variants in the CFTR gene were implicated in four cases and combinations of variants in these three genes were identified in only three cases. The anatomical abnormalities included pancreatic divisum (n = 15), pancreatico-biliary malunion (n = 10) and an annular pancreas (n = 5). Other causes included ascariasis infection, Burkitt’s lymphoma and persistent post-liver-transplant anastomotic strictures, which were three individual risk factors for three patients; two individuals each developed CP from recurrent leaks following pancreatic duct injuries, which were induced blunt non-penetrating abdominal injuries. The co-factors predisposing patients to CP were microvillous inclusion disease, carbamoyl phosphate synthase deficiency and intestinal atresia. Twenty-five patients had ARP, and their aetiologies are listed in Figure 1B.

All individuals underwent pre-procedural imaging (ultrasound, CT and/or MRI). Pre-procedural imaging was reviewed in a multi-disciplinary meeting (paediatric physicians, paediatric and adult surgeons, adult endoscopists, specialist nurses and paediatric and adult radiologists) to confirm the presence of CP or ARP and define the target for HPB endoscopy.

### 3.2. Endoscopic Retrograde Cholangio-Pancreatography

**Figure 2:**
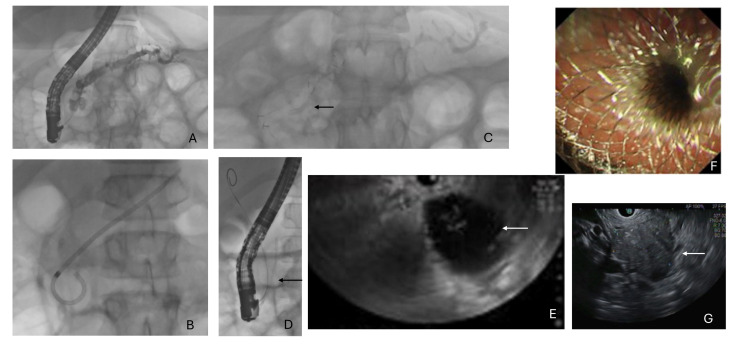
Endoscopic retrograde cholangio-pancreatography (**A**–**D**) and endoscopic ultrasound (**E**–**G**). (**A**) Main pancreatic stricture with a pancreatic duct stone, (**B**) plastic stent, (**C**) fully covered metallic ‘Bumpy’ stent (black arrow), (**D**) Archimedes biodegradable stent (black arrow), (**E**) pancreatic fluid collection (white arrow), (**F**) endoscopic view following successful deployment of 8 × 8 mm LAMS to drain a symptomatic pancreatic fluid collection, (**G**) head of pancreas mass (white arrow) in a patient that underwent a fine needle biopsy and received a subsequent diagnosis of autoimmune pancreatitis.

A total of 222 ERCP procedures were conducted on 98 individuals with CP/ARP (female n = 59, 60%, median age 10 years, range 1–18). Pre-2016, seven individuals underwent a diagnostic ERCP to define their pancreatic anatomy. The targets for therapy included a stricture of the main pancreatic duct (MPD) (Figure 2A) with upstream dilatation (45%), symptomatic stones in the MPD with an associated stricture (65%) or without an associated stricture (20%) (Figure 2A) and an associated lower common bile duct stricture (5%). Strictures of the MPD were commonly seen in the head of the pancreas (70%); 10% were in the neck, 10% in the body and 10% in the tail. A total of 10 individuals underwent an ERCP for acute recurrent pancreatitis (ARP). The median number of procedures was two (range 1–9). Patients had a median of 3 months between procedures (range 1–33). In 22% of cases, only a diagnostic pancreatogram was performed.

Successful pancreatic duct cannulation was achieved in 98% of patients (Table 1). All patients underwent a pancreatic sphincterotomy followed by balloon dilatation (4 mm). A balloon trawl was performed in 80% of cases, followed by the insertion of a single plastic stent (Figure 2B) at the index ERCP in 60% of cases (n = 133). Individuals were planned for a staged upsizing of their stent/the placement of multiple plastic stents or considered for a fully covered metallic stent. A single plastic stent remained in situ for a median of 3.5 months (range 1–17). Twenty-two (88%) patients that underwent ERCP for ARP had a trial of stenting (all plastic). Stricture resolution was more commonly observed with fully covered metallic stents (FcSEMSs) (Figure 2C) compared to a plastic stent alone (75% vs. 51%, *p* = 0.001). The median duration of an FcSEMS was 3.1 months (range 1–5). Patients treated with an FcSEMS (30%) versus a single (50%) or multiple plastic stents (10%) required significantly fewer subsequent procedures to achieve stricture resolution (1.1 ± 0.82 vs. 2.85 ± 0.9 vs. 1.40 ± 0.82, *p* < 0.001). Once the MPD stricture had resolved, an Archimedes biodegradable stent (since 2019) (Figure 2D) was placed in 80% of cases (6 Fr 4 cm, fast degradation, placed in 95% of cases). This avoided the need to remove the MPD stent during a future gastroscopy.

Sixty-five percent of cases (n = 144) were due to an MPD stricture and pancreatic stone. These cases were managed as above, with a pancreatic sphincterotomy and MPD stent placement at the index ERCP, and then were to return for specific stone therapy. A balloon trawl (8.5–12 mm diameter) was the commonest therapy deployed and resulted in ductal clearance in 94% of cases. In one case, ductal clearance was successful with simple flushing only. Ductal clearance was achieved in 81% of the cases requiring a median of two procedures (range 1–6).

No deaths occurred directly relating to the ERCP procedure during the study period. The overall complication rate was 3.6% (n = 8). Specific complications included post-ERCP pancreatitis, PEP (n = 4), the internal migration of the PD stent (n = 1), bleeding (n = 1), perforation (n = 1) and desaturation (n = 1)—all graded as mild/moderate. The patient who developed post-ERCP bleeding required a two-unit transfusion of red blood cells only. All patients with PEP and perforation were managed conservatively (intravenous fluids and analgesia), with no requirement for EUS, IR or surgical intervention.

Patients were followed up with for a median of 24 (0–119) months by local networked paediatric services or by the King’s College Hospital multi-disciplinary paediatric pancreatobiliary team. An improvement in abdominal pain was demonstrated in 76% (n = 74) of the patients who underwent an ERCP. ERCP and plastic stent insertion for ARP resulted in a reduction in hospital admissions (2.5 vs. 1.25, *p* = 0.04). At a median of 24 months, the mean BMI had improved post ERCP (15.5 ± 1.41 vs. 12.9 ± 1.16 kg/m^2^, *p* = 0.001). Twenty percent (n = 19) developed rebound pain requiring reassessment and further ERCP at a median time of 26 months (6–88). Unfortunately, no data were available on whether diabetic control improved in individuals with diabetes. Pancreatic surgery (longitudinal pancreatic jejunostomy) was ultimately required in 3% of individuals (n = 7).

### 3.3. Endoscopic Ultrasound

A total of 54 EUS procedures were attempted in 48 patients. In total, 56% (27) were female and their median age at the time of EUS was 14.5 years (range, 1–18). The commonest indication was for a therapeutic EUS and the drainage of a PFC (65%, n = 35) (Figure 2E). The commonest indication for a diagnostic EUS (n = 19) was the assessment of a pancreatic mass or surrounding lymph adenopathy (20%, n = 11) followed by the assessment of a pancreatic cyst (15%, n = 8). FNB demonstrated features of chronic fibrosis in five patients, inflammation consistent with IgG4 disease in two patients and non-specific inflammatory changes in four patients

In the PFC group (median age 14.5 (6–18) years), seven patients underwent attempted drainage using a plastic Tennenbaum stent (7–10 Fr, 4–7 cm length). Four patients (56%) had a stent successfully placed. In the remaining three, stents could not be placed and instead an FNA was performed. From 2017 onwards, Lumen Apposing Metal Stents (LAMSs) were available and were preferentially used for the drainage of PFC with or without necrosis. In the 25 patients who underwent an EUS-guided cyst-gastrostomy with a LAMS, the stent was successfully placed in all cases. Stent sizes ranged from 8 × 8 mm to 20 × 10 mm (Figure 2F). Three individuals did not undergo stent instrumentation, as their pseudocyst was deemed too small at the time of the EUS procedure. No adverse events were reported at the time of insertion in the LAMS group or the plastic stent group. Four patients (18%) had a recurrence of their PFC requiring a further subsequent LAMS.

The majority of pancreatic masses (Figure 2H) were within the head of the pancreas (64%, n = 7), with the remainder seen within the body of the pancreas. Either a 19 G or 22 G needle was used to perform an FNB. An adequate tissue sample which facilitated an accurate histological assessment was observed in 88% (n = 15 of 19) of these procedures. One patient desaturated during the procedure and another had undergone a previous oesophageal fundoplication procedure, so they could not undergo a sampling of their pancreatic lesion.

Eight patients underwent an assessment for a pancreatic cyst (head, n = 2; body, n = 4; tail, n = 1). Unfortunately, one patient did not tolerate the procedure and, therefore, the procedure was aborted. All successful procedures underwent a fine needle aspiration/fine needle biopsy using a 22 G or 19 G needle to help facilitate a diagnosis.

The overall EUS procedure successful completion rate was 89%. All Hot Axios LAMS were successfully placed. Post-procedural complications were observed in two cases (overall complication rate of 4%) and included sepsis (n = 1) and pancreatitis (n = 1). All were graded as mild and did not require further endoscopic, radiological or surgical procedures. At the time of writing, all stents had been successfully removed at the follow-up gastroscopy. The median follow-up was 27 (1–37) months.

**Figure 3:**
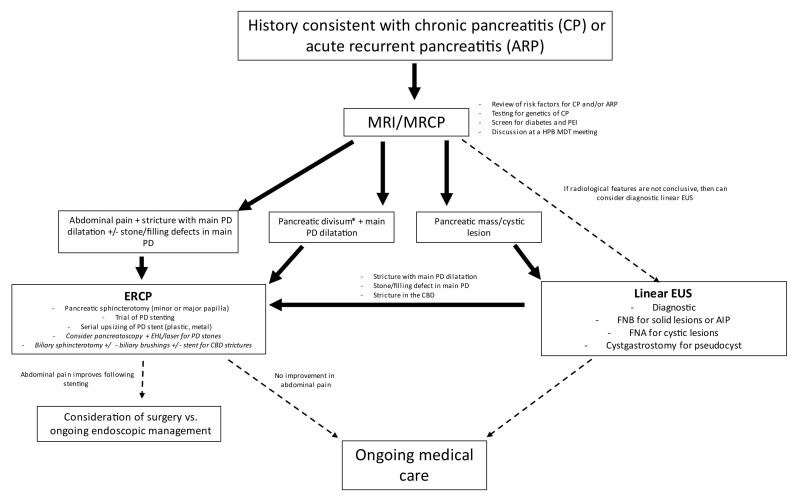
Proposed algorithm for the role of ERCP and EUS in the management of paediatric chronic pancreatitis. CBD, common bile duct; EHL, electro-hydraulic lithotripsy; ERCP, endoscopic retrograde cholangio-pancreatogram; EUS, endoscopic ultrasound; FNA, fine needle aspiration; FNB fine needle biopsy; HPB MDT, hepato-pancreato-biliary multi-disciplinary team; MRI, magnetic resonance imaging; MRCP, magnetic resonance cholangio-pancreatogram; PEI, pancreatic exocrine insufficiency; PD, pancreatic duct. * Other pancreatic anatomical variants include annular pancreas.

Our unit’s algorithm for the management of CP evolved over the study period. A proposed algorithm for the use of ERCP and EUS in the management of CP in paediatric patients is described in Figure 3.

## 4. Discussion

The role of ERCP and EUS in the management of ARP and CP is well established in adult cohorts [7,12]. These minimally invasive techniques with the option of repeat procedures make their endoscopic approach highly attractive in paediatric patients. We have described the largest UK single-unit experience of the role of ERCP and EUS in the management of paediatric ARP and CP. Our data highlight the integral role of ERCP and the increasingly important role of diagnostic and therapeutic EUS.

ERCP outcomes are in keeping with other published data, with a cannulation rate of 98% [6,8,14,15]. Adverse events following an ERCP procedure occurred at a rate of 3.6%. PEP occurred in a total of four patients with no pre-determined high-risk features. During the study period, we saw the introduction and acceptance of rectal NSAIDS as the standard of care in adults, but not all paediatric patients received NSAIDS [16]. We have demonstrated the efficacious use of plastic stents and FcSEMSs, with a superior stricture resolution rate seen with the latter (51% vs. 75%, *p* = 0.001). The use of FcSEMSs is well described in adult cohorts with CP, and their safety and efficacy appear to be similar in paediatric cohorts too [17]. Prospective studies on the use of FcSEMSs in paediatric cohorts would be of benefit. In addition, no adverse events were demonstrated post endoscopy. We demonstrated an improvement in abdominal pain in over two-thirds of the patients who had undergone an ERCP, a reduction in hospital admissions in the patients with ARP and an improvement in BMI. Although we had no data on the improvement of glycaemic control, ERCP interventions undoubtedly resulted in improved patient benefits.

Our study highlights the increasing and evolving role of EUS in paediatrics and, in particular, in the management of paediatric CP. In 2020, European and North American guidelines were published on the roles of EUS and ERCP in the evaluation and treatment of CP [9]. These guidelines are an excellent reference point for centres that are considering undertaking EUS and ERCP in paediatric patients. They highlight the importance of informed consent and appropriate discussions with families pre-procedure and also the areas within the literature where there is a paucity of data, e.g., specific paediatric EUS criteria for diagnosing CP and the need for more efficacy and safety data on therapeutic EUS. Our study reports 35 therapeutic EUS procedures for PFCs. In the majority of these cases (n = 25), a LAMS was inserted successfully. Overall, we reported two adverse events within the group undergoing therapeutic EUS at the time of their index EUS. Although our numbers are small, we feel this contributes to the growing experience and the literature demonstrating the safety of therapeutic EUS in paediatric patients.

This study evaluated the role of EUS and ERCP in paediatric patients with CP. However, there are other indications in paediatric patients for both EUS and ERCP, including the management of biliary structuring disease, EUS-guided liver biopsy, single-operator cholangioscopy and pancreatoscopy, the EUS-guided coiling of gastric varices and EUS-guided portal pressure measurement [18,19,20]. These procedures are all, in theory, possible in paediatric patients and could be used if deemed appropriate.

The advent of new biodegradable stent technology provides a unique opportunity in all patients undergoing ERCP but particularly in paediatric patients [21,22,23]. The Archimedes stent became available for use within our unit in 2019, and the 6 Fr 4 or 6 cm fast degradation stent was adopted as the stent preferred for prophylactic pancreatic stenting in adult and paediatric cases. These stents are also available in 8 Fr and 10 Fr diameters and in a variety of lengths and, therefore, multiple Archimedes stents may be appropriate for use in the management of biliary and main PD strictures [21]. The fast-degradation Archimedes stent was also used in this cohort at the follow-up ERCP once the main PD stricture had resolved. The benefits for paediatric patients of biodegradable stents in the bile duct or PD are clear: the avoidance of a repeat endoscopic procedure and general anaesthetic (GA). Further biodegradable stent technology remains to be evaluated in paediatric patients, namely the Unity B stent [24]. This uncovered, magnesium-based, biodegradable stent which has a ‘skeleton and muscle’ structure has the same mechanical properties as a conventional FcSEMS. The hypothetical benefits here would include, once again, the avoidance of a repeat endoscopic procedure and repeat GA, but also reduced complications related to stent migration (internally or externally down into the GI tract) and improved stricture resolution rates similar to the ‘bumpy stents’ used in this study.

The ERCP and EUS procedures performed in this study were undertaken by adult endoscopists. The patients were referred to and managed by the paediatric liver service in our hospital. We are a high-volume adult HPB centre undertaking approximately 600 ERCPs and 400 EUS procedures per annum. The low complication rate we have reported in this paediatric series is replicated in our adult cohorts. In the last decade, though, more paediatric endoscopists have pursued training in ERCP and EUS [5,9,25]. The volumes required to maintain clinical competence may mean that they may also need to perform procedures in adults. This may be impractical in some centres that are not co-located.

The limitations of this study include its retrospective and single-centre design. In addition, there were non-standardised follow-up data.

In conclusion, our study demonstrates the safe, efficacious and improved long-term benefits of the application of ERCP and EUS in children with CP and ARP as part of a multi-disciplinary approach. Further data and the reporting of clinical experiences are required in terms of the use of new and novel ERCP and therapeutic EUS techniques in paediatric cohorts.

## Figures and Tables

**Table 1:** Outcome data.

**ERCP (n = 222)**	
**Cannulation rates (%)**	98
**Stricture resolution (%)**	63
**- metallic vs. plastic**	75% vs. 51% *
**Improvement in abdominal pain (%)**	76
**BMI pre- and post ERCP**	15.5 vs. 12.9 *
**Complications**	
- Overall (%)	3.6
- Post ERCP pancreatitis (n)	4
- Internal migration of PD stent (n)	1
- Bleeding (n)	1
- Perforation (n)	1
- Oxygenation desaturation (n)	1
- Deaths (n)	0
**EUS (n = 54)**	
**Successful completion (%)**	89
**Complications**	
Overall (%)	4
Sepsis (n)	1
Pancreatitis (n)	1
**Diagnostic (n = 19)**	
**Successful completion (%)**	95
**Complications (%)**	2
- Pancreatitis	1
**Therapeutic (n = 35)**	
**Successful completion (%)**	83
**Complications**	
- Overall (%)	6
- Unsuccessful placement (n)	3
- Sepsis (n)	1
- Pancreatitis	1

ERCP, endoscopic retrograde cholangio-pancreatography; EUS, endoscopic ultrasound * *p* < 0.05.

## Data Availability

Data is not available due to ethical considerations and local R&D policy.
